# Mitochondrion at the Crossroad Between Nutrients and Epigenome

**DOI:** 10.3389/fendo.2019.00673

**Published:** 2019-10-04

**Authors:** Giusi Taormina, Antonio Russo, Mario A. Latteri, Mario G. Mirisola

**Affiliations:** Surgical, Oncological and Stomatological Disciplines Department, Medical School, University of Palermo, Palermo, Italy

**Keywords:** mitochondria and metabolism, nutrition and epigenome, calorie restriction and epigenome, FMD and epigenome, nutrients and epigenome

## Abstract

Epigenetic profile is the link between the regulation of nuclear gene expression and the environment. The most important factors capable of significantly affecting the cellular environment are the amount and quality of nutrients available. Mitochondria are both involved in the production of some of the molecules capable of directly affecting the epigenome and have a critical role in the conversion of nutrients into usable energy. Carbohydrate and fats are converted into ATP, acetyl-CoA, SAM, and NADH. These high-energy substrates are, in turn, capable of driving the epigenetic profile. We describe substances capable of affecting this mechanism. On the other hand, nutritional interventions capable of reducing calories or significantly impairing the normal Acetyl-CoA production or the SAM-SAH ratio also impact chromatin methylation and histone modification, suggesting a critical role of mitochondria on nutrient-dependent epigenetic profile.

## Introduction

**Figure 1:**
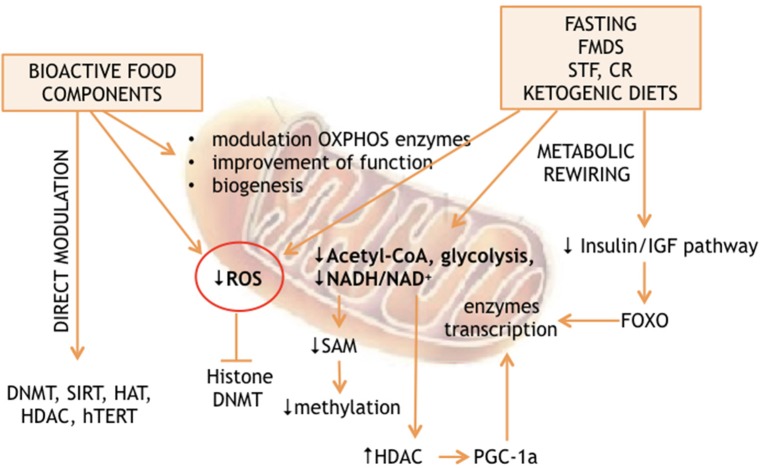
Effect of nutrients and diets on mitochondria and epigenetic.

The analysis of the non-genetic factors network, capable of affecting the lifespan of living organisms, revealed nutrition as a major determinant of longevity. On the other hand, many studies found the environment to be an epigenome driver. The predicted existence of a link between nutrition-dependent modulation of longevity and environment-dependent reprogramming of epigenomes has also been demonstrated (1–5). Recent advances suggest that nutrition can affect the epigenome through 2 general mechanisms: either directly, thanks to substances, which interact with the enzymes responsible for “writing” or “erasing” the epigenetic profiles; or indirectly through metabolic rewiring. The latter can be induced by calorie restriction, fasting, fasting mimicking diet, time restricted feeding, or ketogenic diet. These extreme diets are capable of producing massive cellular reprogramming via partially unclear molecular cascades. Interestingly, both mechanisms converge on mitochondria, which produce many factors and substrates essential for epigenetic modifications and are at a crossroads of cellular energy metabolism (Figure 1).

## Nutrient Capable to Directly Affect the Epigenome

Choline, methionine, and folate (see Table 1) deficiencies are responsible for DNA hypomethylation (6) in several species including humans. During embryogenesis, folate has a critical role in the establishment of the epigenome, while in adults, folic acid deficiency is linked to the development of several cancers, such as brain, lung, colorectal, cervix, ovary, and breast cancer (7, 8). On the contrary, a diet rich in folate, vitamins B12, B2, and B6, increases methylation of L1s sequences (long interspersed nucleotide elements) in peripheral blood mononuclear cells, resulting in a decreased risk of developing cervical intraepithelial neoplasia in humans (9). Folate, choline and methionine metabolism are part of the universal metabolism known as one carbon metabolism (1-C). It has an essential role in nucleic acid, methionine and serine biosynthesis. Enzymes of the 1-C metabolism are present in both cytosol and mitochondria with specific roles. While the enzymes and cofactors responsible for homocysteine re-methylation to methionine are localized into the cytosol, the mitochondrial 5,10-methylene-THF dehydrogenase links the 1-C metabolism to NADH availability (10). The observed crosstalk between cytosol and mitochondria is strengthened by the observation that respiratory chain deficiency triggers the expression of serine synthesis genes, thus feeding the 1-C metabolism (11, 12). It must also be noted methionine is an essential amino acid, but it is also the major methyl donor molecule through its adenosylated product *S*-adenosylmethionine (SAM). SAM, the reactive methyl carrier, is second only to ATP as an enzymatic cofactor for cellular abundance and has a critical role in epigenome establishment and maintenance (13, 14). Interestingly, the effect of folate deficiencies may be reverted by its supplementation. For example, the hypermethylation of the PPARα promoter, induced by folate deficiency during pregnancy, can be reversed by folate supplementation in neonate rats and sheep (15).

**Table 1:** Bioactive foods and their role on epigenenome and mitochondria.

**Bioactive food component**	**Food source**	**Epigenetic functions**	**Effects on mitochondria**
Curcumin	*Curcuma longa*	SIRT1 activation, H3 and H4 acetylation, DNMT1 inhibition, HAT, and HDAC inhibition	↓ mtROS Mitochondrial biogenesis Mitophagy
Genestein	Soybeans	DNMT inhibition, DNA methylation	Modulation of enzymes ↑ Mitochondrial mass Improvement of mitochondrial function
Resveratrol	Berries, peanuts, grapes, wine	SIRT1 regulation, alteration of histone acetylation, FOXO deacetylation	Mitochondrial biogenesis ↑ Mitochondrial mass
Folate	Legumes, eggs, leafy green vegetables, beets, citrus fruits, Brussels sprouts, broccoli, beef liver, cereals	DNA methylation	↑ NADPH Mitochondrial biogenesis
Methionine	Animal proteins	DNA methylation	↓ mtROS ↑ Cox I activity ↑ Mitochondrial respiration
Choline	Salmon, eggs, meats, shrimp, broccoli, green peas	SIRT3 activation	UPRmt induction Modulation of ketone body and fatty acid metabolism
Lycopene	Tomatoes	DNA methylation	Improvement of mitochondrial function ↓ oxidative damage
Sulforaphane	Cruciferous vegetables	↓ DNMT1/3 expression, ↓ HDAC, hTERT inhibition	Improvement of mitochondrial function Mitochondrial biogenesis

Finally, diet methionine concentration can affect the ratio between s-adenosyl methionine and s-adenosyl homocysteine (SAM/SAH), impacting methylation reaction both on DNA and on histone proteins of rodents and humans (16).

Resveratrol (3,5,4′-trihydroxy-trans-stilbene, see Table 1) is the most studied anti-aging compound and is involved in histone modification and DNA methylation. Resveratrol supplementation increases longevity in simple model organisms and in mammals (17, 18). At the molecular level, it regulates SIRT1 (19, 20) and FOXO deacetylation affecting cells survival (21). The major *in vivo* effects are increased insulin sensitivity (22), anti-inflammatory effects, inhibition of NF-κB, thus inhibiting the development of cancer in cellular models of breast and prostate cancer (23, 24). Resveratrol has many interconnections with mitochondrial metabolism. Its supplementation increases mitochondrial mass (25, 26). SIRT1-dependent deacetylation of PGC-1α results in biogenesis of mitochondria (27, 28), which in turn co-activates the nuclear respiratory factors (NRF-1 and NRF-2), resulting in mitochondrial biogenesis (29). There isn't a general agreement on the mechanism by which resveratrol acts but some hints suggest the involvement of mitochondrial complex III (30–33). Alternatively, AMPK could be the link between resveratrol and SIRT1 through NAD+ concentration modulation (34, 35). SIRT1 is also activated by quercetins, and catechins in different model systems (36, 37).

Curcumin is a polyphenol extracted from the spice Curcuma longa, known as a natural anti-inflammatory agent. Dietary curcumin reduces mitochondrial ROS production (see Table 1) and improves markers of aging in wild-type mice. It is involved in different epigenetic modifications: it regulates H3 and H4 acetylation, DNMT1 and, with a mechanism that involves miRNA, SP1, and PTEN. Its most relevant effect is the inhibition of NF-κB (38). In mice it prevents heart-failure through HATs inhibition, HDACs and p300 degradation induction (39, 40). Interestingly, a homozygous deletion of the mitochondrial uncoupling protein 2 UCP2–/– reverses this effect in mice (41), thus suggesting the active role of mitochondria in this mechanism. In a rat model and in endothelial cell cultures, nitric oxide synthase (eNOS), AMPK phosphorylation, upregulated UCP2, and reduced ROS production are observed after curcumin supplementation, while inhibitors of either AMPK or UCP2 abolish the curcumin effect (41).

Tea polyphenols and catechins are scavengers of free radicals and singlet oxygen. Their efficacy in the prevention and treatment of many diseases has been demonstrated. They affect apoptosis and provoke cell-cycle arrest in human cancer cell lines (42). Epigallocatechin prevents UV-induced carcinogenesis of the mouse skin (43), while epicatechins and catechins have shown anti-aging effects in *C. elegans* (44).These substances have many epigenetic targets: H3 and H4, NF-κB, IL-6, SUZ12/HAT, HDAC, HMT, P16INK4a, RNRβ, RECK1, hTERT, WIF-1, RXRα, RXRβ, CDX2/DNMTI, and Bcl-2. Epigallocatechin binds to the catalytic region of DNMT1 and inhibits its activity (45). Furthermore, it leads to a decrease in DNMT1, DNMT3a, DNMT3b, and HDAC levels, whereas, it increases the acetylation of histones H3 and H4 at specific sites (46). It has recently been demonstrated that isoflavones stimulates mitochondrial biogenesis and improves mitochondrial function in diabetes, chronic heart failure and renal injury, as well as in aging of rodents (47–49).

Suphorafene induces cell-cycle arrest and apoptosis in mice cancer cells by epigenetic mechanisms (2, 17, 50, 51). A similar effect has been detected for lycopene (52), quercetin (53), and ellagic acid (contained in pomegranate, walnuts, and almonds) (54). Low doses of suphorafene are related to hTERT inhibition and to the reduction in DNMT1 and DNMT3a expression levels (16, see Table 1). Furthermore, suphorafene determines deacetylase down-regulation in melanoma cells *in vitro*, inhibiting their growth and proliferation (55). Interestingly, sulphoraphane has been demonstrated to enhance ROS and mitochondrial membrane depolarization in human ovarian cancer cell lines (56).

Genistein, contained in soybeans, take part in modulation of chromatin structure and DNA methylation; histones SIRT1, p21, p16, PTEN, p53, FOXO3A, and hTERT are its epigenetic targets (57, 58), which, in turn, are key regulators of cell-cycle regulation and cell survival. A recent study shows that supplementing Laying broiler breed hens with genistein can alter lipid metabolism in the offspring chicks through epigenetic modifications that upregulate PPARδ expression, improving antioxidative capability and growth performance (59). In addition, genistein induces in mitochondria, and modulation of enzymatic activity of components of the oxidative phosphorylation system (see Table 1).

## Nutrients Indirectly Affecting the Epigenome

Calorie restriction and the ketogenic diet, as well as fasting and fasting-mimicking diets are nutritional interventions capable of significantly affecting longevity in a wide range of living organisms. The ability of CR to modify the epigenome is suggested by many observations. CR protects against age-related DNA methylation changes as described in different mammalian tissues: Kidney (60), blood (61), liver (62), hippocampus (63), and cerebellum (64) being the most affected in mice and rats. Expression studies of genes coding for proteins involved in mitochondrial function revealed increased expression of PGC1α, TFAM, eNOS, SIRT1, and PARL genes after 6 months of calorie restriction in humans (65). In addition, PGC-1α activation by NAD+-dependent Sirt1 deacetylation connects OXPHOS and calorie restriction (66, 67). PGC-1α and the other two members of this gene family are transcriptional activators (PGC-1α, PERC, and PGC-1β), which affect the transcription of genes involved in mitochondrial energetics modulating thermogenesis, fat oxidation and mitochondrial biogenesis (68, 69). Assumption of very low amount of carbohydrate with high or adequate protein content (ketogenic diets) will force the body to produce, mainly in the liver, ketone bodies which can be subsequently used by many extra-hepatic tissues (e.g., nervous system) as energy sources in place of sugar. This condition can also be transiently triggered by fasting or the fasting-mimicking diet and possibly by calorie restriction. The ketogenic diet has been proposed as an adjuvant therapy for cancer patients based on the idea that most of the cancer cell metabolism is based on high rate glycolysis (known as the Warburg effect). Normal cells are also capable of using glucose as an energy source but can also rely their metabolism on the ketone bodies produced in the liver tissue when glucose concentration is scarce. On the contrary, cancer cells have lost this ability and base their biosynthetic pathways on glucose only. The reduced glucose availability will also provoke a reduction in circulating insulin. Therefore, the insulin dependent (IRS)/RAS/RAF/MEK/MAPK and the RAS/PI3K/AKT/mTOR transduction cascades are turned off (70). This causes downstream reduction of phosphatidylinositol triphosphate (PIP3) production, PI3K and AKT, as well as a drop in MAP kinase activity, and unphosphorylated forkhead box O transcription factor (FOXO) is forced to remain in the nucleus. Nuclear FOXO acts as transcription factor promoting the transcription of hundreds of genes, including mitochondrial glutathione peroxidase, superoxide dismutase 2, and catalase.

Even though chronic calorie restriction delays the growth of many cancers in mice, it imposes weight loss and possibly also immunosuppression, making it infeasible for cancer treatment. In addition, the avoidance of malnutrition is not an easy task for most of the subjects under calorie restriction. On the contrary, complete fasting or a low calorie diet capable of mimicking the effects of fasting (fasting mimicking diets, FMDs) consecutively for 2–7 days have a profound effect on cellular metabolism and cause only a transient loss of weight. Re-feeding is in fact normally accompanied with weight regain unless it is otherwise desired. Fasting for 2–4 days, known as Short Term Fasting (STF), is capable of inducing multiple metabolic changes, blood sugar reduction, drop of circulating IGF1, increased IGF1BP/IGF1 ratio and transient ketone bodies increase. From a metabolic point of view, these nutritional interventions all have the induction of ketosis in common. Many conditions are capable of forcing Ketone bodies oxidation to become a significant part of the mammalian energy metabolism, including diets with very low carbohydrate, strong endurance exercise without carbohydrate supply, fasting, starvation, the neonatal period, pregnancy, and likely calorie restriction. The concentration of ketone bodies normally ranges from 100 to 250 μM in healthy adults and rises up to ~1 mM after prolonged exercise or 24 h of fasting. A higher concentration of ketone bodies is observed in some pathological conditions like diabetic ketoacidosis, where ketone bodies can rise up to a harmful 20 mM (71–75). The mammalian liver uses acetyl-CoA to produce most of the circulating ketone bodies, which are then used by extrahepatic tissues for terminal oxidation (71, 74, 76). Ketone bodie's metabolism is linked to cytoplasmic and mitochondrial pathways. β-oxidation (FAO), gluconeogenesis, the tricarboxylic acid cycle (TCA), *de novo* lipogenesis (DNL), and sterols biosynthesis are just examples of pathways interconnected with the availability of ketone bodies. Excessive production of acetyl-CoA by β-oxidation and/or depletion of oxalacetate are both capable of triggering liver ketogenesis. The rate of ketogenesis is not linearly dependent on hepatic acetyl-CoA concentration (77, 78). However, hepatocytes aren't capable of metabolizing the ketone bodies that they produce. Heart, brain and skeletal muscles can instead rely on ketone bodie's metabolism, converting them to Acetyl-CoA, which in turn fuels TCA cycle for terminal oxidation. Alternatively, ketone bodies can feed sterol synthetic pathways as well as lipogenesis or can be excreted in the urine (79–81). The amount of acetyl-CoA available can modulate histone acetylation, since this molecule is also the cellular acetyl donor used during histone acetylation. Increased circulating acetyl-CoA, which is directly dependent on the rate of its production, is in fact related to H3K9 acetylation on a genome-wide analysis. It appears that acetyl-CoA availability can force cells to enter growth through histone acetylation and consequential transcription of pro-growth genes (82). In yeast, it has been clearly demonstrated that acetyl-CoA availability is metabolically sufficient to trigger histone acetylation (82). Thus, what fasting, fasting mimicking diet and possibly calorie restriction, as well as ketogenic diet, all have in common is a reduced availability of acetyl-CoA, which results in epigenetic anti-growth modification. However, the energy reduction consequential to fasting or the reduced glycolysis, due to both fasting or ketogenic diet, also affects the available reducing equivalents. NADH will, in fact, be oxidized to NAD+. The latter is a substrate for the class III histone deacetylases, which leads to DNA-binding protein deacetylation with a consequential increase in chromatin positive charge leading to transcription repression and growth arrest.

Energy availability and specific nutrient supply modification, such as those obtained by calorie restriction or by fasting and fasting mimicking diet (see Figure 1), can also modulate nuclear gene expression through DNA or histone methylation by S-adenosyl-L-methionine (SAM). In the cytosol, L-methionine + ATP can give SAM + Pi + PPi. Thus, energy production is linked to methylation of lysine and arginine, as well as cytosine DNA by SAM as already described above. On the other hand, methionine can be obtained by homocysteine methylation in a process regulated by mitochondrial metabolism. The mitochondrial and cytosolic one-carbon metabolisms are interconnected through the exchange of serine and glycine, which are modified in both compartments by the activity of methylene-tetrahydrofolate, thanks to the cytosolic and mitochondrial serine hydroxymethyltransferases. However, methylene-tetrahydrofolate is converted to formyl-tetrahydrofolate in mitochondria, forcing the 1 carbon metabolism to produce formate instead of serine into the mitochondria, which is in turn used for purine biosynthesis. Alternatively, serine is exported to the cytosol. The cytosolic one-carbon metabolism uses serine to convert homocysteine to methionine through methylene-tetrahydrofolate and methyl-tetrahydrofolate, which can then be used for SAM production. Within mitochondria, the direction of the synthesis toward serine or formate production depends on NAD+ availability. Inhibition of OXPHOS increases the NADH/NAD+ ratio, inhibiting the production of methylene-tetrahydrofolate and thus serine, methionine and SAM production (83), while reintroduction of mtDNA into cancer cells is capable of reestablishing the wild type methylation status (83, 84), thus confirming the strict relationship between mitochondrial function and the epigenome profile (Figure 1).

## Conclusion

We have briefly reviewed the interconnections between mitochondria and epigenetic profile from the nutrients point of view. It is now becoming clear that epigenome has a critical role in cellular metabolism and that its drift may contribute to the aging process. Since the efficiency and number of mitochondria may be addressed by pharmacological and dietary approaches (85–87), we believe that mitochondria may be a potential target for preventive medicine by dietary, as well as supplemental treatment, and for next-generation anti-aging drugs.

## Author Contributions

GT and MM wrote the manuscript. AR, ML, and MM revised the manuscript.

### Conflict of Interest

The authors declare that the research was conducted in the absence of any commercial or financial relationships that could be construed as a potential conflict of interest.
